# Changes in the Brain Activity and Visual Performance of Patients with Strabismus and Amblyopia after a Compete Cycle of Light Therapy

**DOI:** 10.3390/brainsci11050657

**Published:** 2021-05-18

**Authors:** Danjela Ibrahimi, Jorge D. Mendiola-Santibañez, Enoé Cruz-Martínez, Alfonso Gómez-Espinosa, Irineo Torres-Pacheco

**Affiliations:** 1 Facultad de Medicina, Universidad Autónoma de Querétaro, Santiago de Querétaro 76176, Mexico; 2 Facultad de Ingeniería, Universidad Autónoma de Querétaro, Santiago de Querétaro 76010, Mexico; irineo.torres@uaq.mx; 3 Campus Querétaro, Universidad del Valle de México, Santiago de Querétaro 76230, Mexico; 4 Hospital Infantil Teletón de Oncología, Anillo Vial Junipero Serra 1999, Santiago de Querétaro 76140, Mexico; enoecruzmtz@gmail.com; 5 Escuela de Ingenieria y Ciencias, Tecnologico de Monterrey, Av. Epigmenio González 500, Fracc. San Pablo, Santiago de Querétaro 76130, Mexico; agomeze@tec.mx

**Keywords:** light therapy, brain activity, quantitative electroencephalography, strabismus, amblyopia

## Abstract

This research assesses the brain activity and visual performance at baseline and after light therapy (LTH), of seventeen patients with strabismus and amblyopia (SA), and eleven healthy controls (HCs) from Querétaro, México. Quantitative electroencephalogram analysis (qEEG) was used to record the brain activity, and clinical metrics such as the visual acuity, angle of deviation, phoria state, stereopsis, and visual fields determined the visual performance. Results showed a constant higher alpha-wave frequency for HCs. Low voltages remained negative for HCs and positive for SA patients across stimulation. After LTH, high voltage increased in SA patients, and decreased in HCs. A second spectral peak, (theta-wave), was exclusively recorded in SA patients, at baseline and after LTH. Positive Spearman correlations for alpha-wave frequency, low and high voltages were only seen in SA patients. Synchronized brain activity was recorded in all SA patients stimulated with filters transmitting light in the blue but not in the red spectrum. Enhancement in the visual performance of SA patients was found, whereas deterioration of the phoria state and a decrease in the amount of stereopsis was seen in HCs. To conclude, only a suffering brain and a visual pathway which needs to be enabled can benefit from LTH.

## 1. Introduction

The brain is the body’s most complex organ, and has been the object of study of the neuroscientific community for decades [1]. Nonetheless, there is a lot to be discovered about the brain network organization, its connections, and functions. Researchers have shown that the brain activity and its neuroplasticity can be enhanced with energy-based stimulation, including light, sound, and movement [2]. Even though LTH has been successfully used in the field of neuroscience [3,4], this is the first paper to assess the afterwards effect of exposure to LTH in SA patients using quantitive electroencephalography (qEEG) analysis. The aim of this study is to measure and analyze the impact of a complete cycle of LTH in SA patients and propose its use as an essential element of the whole therapy treatment to achieve brain synchrony, and enhance the visual performance of these patients. The visual system dominates over the rest of sensorial modalities, if we consider that 70% of the total sensory input to the brain comes from the two eyes. Brain research has identified 300 intracortical pathways linking over 30 different cortical areas involved in processing visual information. Taking into consideration that 50% of the cortex is related to visual processing, the treatment of visual deficiencies becomes crucial [5]. Patients with strabismus and amblyopia were chosen for this study as both visual conditions have been related to abnormal development of the visual system, in particular by affecting binocularity and three-dimension perception of the world [6]. These visual deficiencies are followed by first and second-order abnormalities processing of visual information [7], as well as changes at deeper cortical levels [8]. Substantial variations in brain activity patterns [9], cortical thickness [10], and functional connectivity [11], evidence that strabismus and amblyopia come with changes in the white and gray matter, depending on the type and time of appearance [10,11,12,13]. When the dorsal and ventral pathways are affected as occurs in SA patients, visual judgment, visual attention [9,14], memory and learning aspect can be altered as well [15]. Likewise, the presence of theta-waves at baseline, on different brain regions and particularly on frontal lobes, suggests that strabismus and amblyopia are part of a neurodevelopment disorder [16], which requires deeper attention and intervention. The results published in our previous paper focused on the cortical electrical activity of SA patients at the precise moment of receiving a light stimulus [16], but this research goes a step forward and analyzes the permanence of the light effect on the brain activity of SA patients after a complete cycle or twenty consecutive sessions of LTH, once the stimulus is off [17,18], as well as its influence on the visual performance of these patients. A control group was used as a comparative to SA patients across the light therapy program. qEEG was used to measure the impact of light therapy (LTH) on the brain activity of SA patients and healthy controls (HCs) in the waking-state. qEEG was chosen for this research as it allows obtaining a topographic brain mapping, looking for focal alterations, as well as identifying bands (frequencies) with greater precision, and detecting one or more spectral peaks [19,20]. This way, qEEG determine the electrical activity in different brain regions and monitor the symmetry of alpha-wave activity within hemispheres, which relates to the functionality of the human brain [21]. Light, on the other hand, has been demonstrated to impact both visual and non-visual processes, including retinal functions, circadian rhythms, metabolic processes, sleep, mood, and growth. Cognition can be enhanced by modifying its intensity and wavelength [22], and therapeutic effects can be triggered in patients with visual dysfunctions [17,18], and degenerative diseases [3,4]. Specifically, in our study, visible light was used for the treatment program, as cells responsive to luminance, color, or luminance and color, were found in the primary visual cortex (V1) [23], whose neural brain activity and synchronization was influenced and modified by its different wavelengths [24]. Eye muscle surgery, eye patching and visual therapy have been used to treat strabismus during decades. However, eye muscle surgery and eye patching are frequently accompanied by unwanted outcomes. Regression, consecutive strabismus and amblyopia of the patched eye are often presented in these situations. Visual therapy on the other hand, could last from 2 to 3 years, and results are not always maintained once the therapy is over. Additionally, literature feature few information about the brain activity of SA patients before and after the visual therapy program. LTH, has proven to bring synchronicity between hemispheres, which is the key to a healthy functional brain network. LTH then should be the first step before starting any other treatment to enhance brain activity and prepare the shore for the next step. A balanced brain guarantees better and more stable results through the time [25,26]. Our research would make a significant contribution in the field of visual health and neurodevelopment disorders, as it broaden the understanding of the LTH effect across the brain of patients with strabismus and amblyopia, and the degree at which it can be clinically reflected in the visual performance and how to enhance it. Evidence-based decisions can then be taken to determine the extent of LTH required for the whole-therapy treatment of a patient with strabismus and amblyopia or other groups of patients with neurodevelopmental disorders.

## 2. Neuronic™

**Figure 1:**
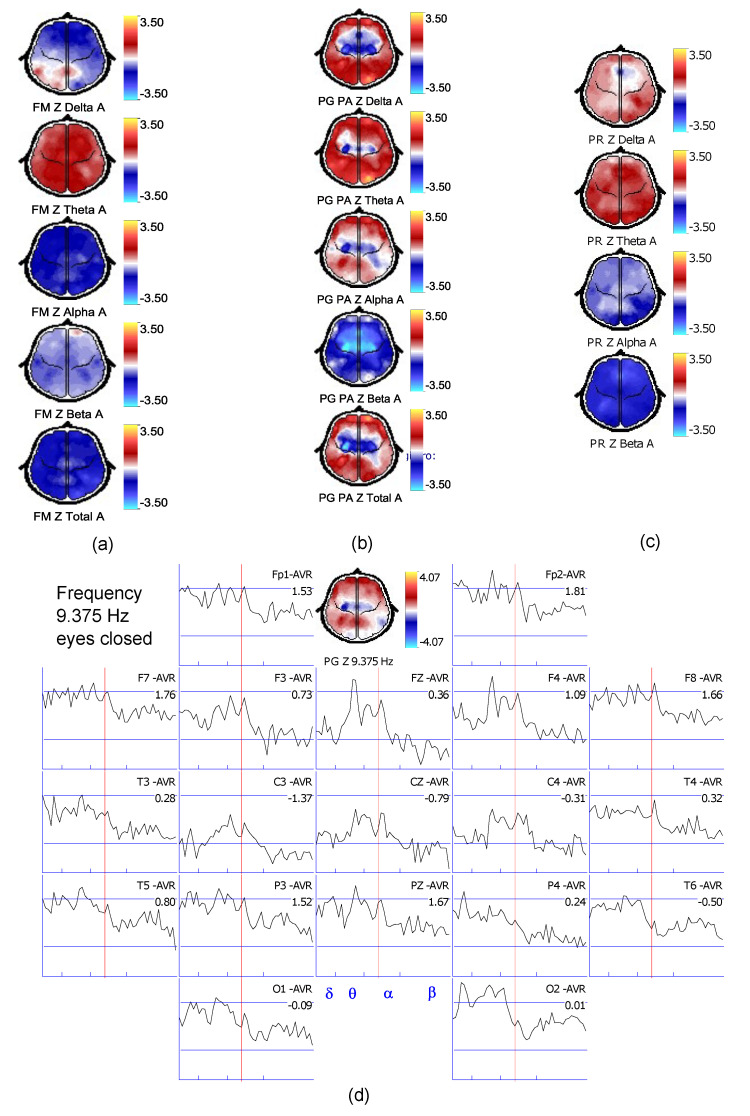
Images obtained from Neuronic software after the LTH program. (**a**) the mean frequency; (**b**) absolute power; (**c**) relative power; (**d**) FFT result.

Neuronic is a company developing technology to perform EEG quantitive analysis (qEEG) which examines the electrical activity of the brain. qEEG or digital brain mapping (DBM) is the mathematical processing of digitally recorded EEG to highlight specific waveform components. qEEG estimates spectral activity at the electrodes (topography) as well as at the sources (tomography). The methods use EEG spectral analysis through Fast Fourier Transform (FFT) [21], producing quantitive data which can be statistically analyzed. Figure 1 represents broad and narrow band measures through qEEG using FFT. Specific technical details about the qEEG analysis using Neuronic have already been published [16].

## 3. Materials and Methods

A total of seventeen patients with strabismus and amblyopia participated in this study, of whom 47% were female and 53% male. 47% presented esotropia, one of whom also had hypertropia/hypotropia as a secondary deviation. 41.2% suffered from exotropia, three of whom presented hypertropia/hypotropia as well. Only one had pure hypertropia/hypotropia and another one had anisometropic amblyopia. Moreover, 41.2% presented stereopsis. One patient had gross stereopsis, and the other six presented fine stereopsis, which affected the standard deviation value. Of the seventeen patients, 35.3% had left-eye motor dominance, and 64.7% had right-eye motor dominance. All patients were right-handed. Additionally, eleven healthy controls (HCs) were matched with the patients in terms of age, sex and economic status, of whom 54.5% were male and 45.5% female. All HCs patients presented orthophoria at far and exophoria at near (mean 12.27 ± 5.69 diopters). Of the eleven patients, ten were right-handed and one was left-handed. Nine of the eleven patients had right-motor dominance and two left-motor dominance (see Table 1). The small number of HCs was due to the time frame of the LTH program. The hypothesis of the present research is that LTH should produce changes in the brain activity of SA patients represented by the alpha-wave distribution, anteroposterior gradient, and the interhemispheric synchronicity. These changes should be reflected in the visual performance of SA patients measured through the amount of the visual acuity, degree of stereopsis, angle of deviation and dynamic visual fields. The protocols were approved by the Ethics Committee of Autonomous University of Querétaro with approval number 10,848, and conform to the principles of the Declaration of Helsinki. Written informed consent was obtained from the participants or their parents before their enrollment in the study.

### 3.1. Inclusion Criteria

Diagnosis of primary strabismus and amblyopia; best-corrected visual acuity of ≥0.7 logMAR; age of 8–30 years; IQ score in the norm for their chronological age, as reported by their schools and confirmed by their medical histories.

### 3.2. Exclusion Criteria

Diagnosis of secondary strabismus (neurological, traumas, ocular pathologies) and/or a history of vision therapy; previous eye surgeries, dissociated and consecutive strabismus; photosensitivity; the presence of conditions such as attention-deficit/hyperactivity disorder, epilepsy, dyslexia, or depression; the use of medications that could affect the central nervous system (CNS); and premature birth. In addition, all HCs met the following criteria: (i) no history of eye disease, (ii) best-corrected visual acuity (VA) ≥ 0.2 logMAR units, (iii) no history of any neurological condition, nor psychiatric disease, (iv) no use of medications that could alter the CNS. Data on the medical histories of the patients and results from their clinical examinations were collected at the Autonomous University of Querétaro, México, from August 2019 to August 2020. Eligibility was established based on the visual clinical evaluation process presented as follows.

### 3.3. Data Collection

Data collection was divided in five phases:

For better results, the visual performance, EEGs and the LTH program were performed from 10 am to 12 am and participants were recommended to maintain a constant sleep cycle which included 8–9 h of sleep per day.

**Figure 2:**
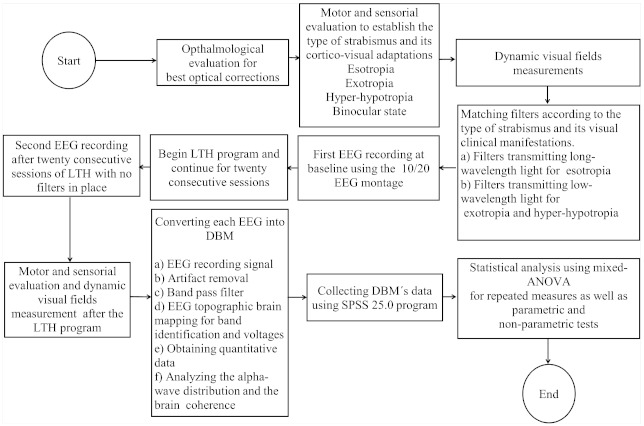
The flow diagram illustrates the steps followed in this research.

Phase one: Detailed medical histories to establish the cause of strabismus and amblyopia were collected. Near and distance visual acuity and noncycloplegic objective refraction were performed. Cycloplegic objective refraction using two drops of 1% tropicamide [27], and ophthalmoscopy to establish the type of fixation under the cycloplegic effect was carried out.
Phase two: Subjective refraction for the best optical correction was performed. Posteriori, the visual efficiency evaluation was carried out. It included near and distance visual acuity with the new prescription; measurements of the deviation and magnitude of strabismus; motor and sensory fusion; fixation and correspondence using the Macular Integration Test and Bagolini lenses; motility (paresis and paralysis) [28]; pupillary reflex; hyper-hypotropia; and the assessment of dissociated elements such as latent or manifest nystagmus, dissociated vertical deviation, angle variability, and limitation in abduction followed by horizontal incomitancies [25,29]. Patients with a visual acuity of ≤0.2 logMAR were reexamined after wearing the newly prescribed glasses for four weeks [30]. The type of strabismus was established based on the clinical data collected. Visual field measures were performed using the Functional Color Field Tester (FCFT) devised by the Bernell Corporation with the best optical correction. Except for visual fields measures which are explained below, the above-mentioned clinical testing for the visual performance of participants can be find in [16].
  Visual field measures were performed in monocular and under scotopic conditions, using the optical correction of the patient. For this purpose, a $19^{\prime \prime }$ screen with a minimum resolution of 768–1024 as recommended by the FCFT manufacturers was used. The movement perception (white target), the functional visual fields (red-blue-green targets), and the blind spot were evaluated through the FCFT. The estimators of the parameters were as follows: diameters of 3.2 and 1.6 mm for the white target and the red, blue, and green targets, respectively. The target-presentation speed was of 36 mm/s; target brightness setting of 176 (no unit), and a random order of presentation regardless of target color. Targets were initially presented at 15 degrees from the center of the screen. The central fixation target was a single digit that randomly flashed at intervals of 1500 ms. Visual field measures were obtained before beginning any treatment (basal), after eight treatment sessions (to monitor progress), and after 20 sessions of LTH program (final) [17,18].
Phase three: Only patients who met the inclusion criteria were scheduled for the baseline brain activity measurement through electroencephalography (EEG), and the subsequent LTH program. Filters were matched according to the type of strabismus and the visual clinical manifestations as shown in the flowchart in Figure 2 which illustrates the steps followed in this research.
Phase four: A second EEG recording after twenty consecutive sessions of LTH was performed. Visual performance and visual field measures were repeated after the complete cycle of LTH treatment.
Phase five: qEEG data were obtained and the statistical analysis using the SPSS 25.0 program was performed.

### 3.4. EEG and LTH Parameters and Procedure

The international 10/20 EEG montage [31] was used to record the brain electrical activity of participants. The evaluation was performed under low-light conditions in both recordings. Data were recorded in the Neuronic™ Psychophysiology system. Two EEG recordings were performed for each patient: before (at baseline) and after twenty sessions of LTH (a total cycle of light therapy program) [17].

The LTH accommodation using the visible spectrum (380–780 nm) is illustrated in Figure 1 [17]. The visible spectrum (380–780 nm), was used for LTH. Of a set of 13 different glass filters of 24 mm in diameter and between 4 and 8 mm in thickness which transmit light in the blue and red spectrum were available, two or three were used in combination and mounted near the bulb according to the needs of each patient. Filters were chosen based on the patient’s medical history, symptoms, and clinical findings according to the protocol for patients with strabismus and amblyopia established by the College of Syntonic Optometry (CSO). Filters transmitting light in the blue spectrum were used for exotropia and hyper-hypotropia, whereas filters transmitting light in the red spectrum for esotropia. For HCs, the spectrum of the filter used, was randomly chosen. Light stimulation was administered in a 20-min session for all participants during 20 consecutive days. The Syntonizer of the CSO used for light stimulation features the following characteristics: a black tube of 50 cm in length, a frosted lens of 55 mm in diameter that appears as a glowing dot with saturated color, and a 115- V bulb with a vibration series 50-W that delivers 1.4 Lux when unfiltered. The light could be presented as steady or strobed, as proposed by CSO [17,18]. More details related to the EEG procedure and LTH program, can be find in [16].

### 3.5. Statistical Analysis

Mixed ANOVA for repeated measures was used to analyze and compare the mean differences of the data collected through the qEEG, between SA patiens and HCs at Time 1 (at baseline) and Time 2 (after 20 sessions of LTH). Spearman correlation was used to assess the relationship between two variables using a monotonic function. Adjustment for multiple comparison was performed using Bonferroni. The normality of data distribution was checked with Shapiro-Wilk (S-W) test. For the visual performance, T-paired test and Wilcoxon test were used to detect changes between two related samples based on the normality of data distribution.The level of statistical significance is expressed as a *p*-value between 0 and 1. All statistical analyses were performed with SPSS Statistics Base 25.0. The confidence level (CI) used in this study was 95%, with an alpha of 0.05 (α = 0.05).

## 4. Results

### 4.1. Demographic and Visual Measurements at Baseline

No significant age differences (*p* = 0.49) were detected between the two groups. By contrast, the differences observed between the two groups in the best-corrected visual acuity of both eyes at far (*p* = 0.001) and near (*p* = 0.004 and 0.002 for OD and OI respectively) and the amount of stereopsis (*p* < 0.001) were statistically significant (Table 1).

### 4.2. qEEG Measurements

A 2 (Time) × 2 (Groups) mixed-model ANOVA for alpha-wave, revealed that the main effect for Groups was statistically significant F (1.26) = 23.03, *p* < 0.001, Eta-squared = 0.47 (Table 2). Thus, there was a significant overall difference in its value when SA (m = 9.62) was compared to HC (m = 11.20) group (Table 3). A significant main effect for Time was also obtained, F (1.26) = 4.54, *p* = 0.043, Eta-squared = 0.15 (Table 4). Alpha-wave values after LTH (m = 10.59) were higher than at baseline (m = 10.21) (Table 5). However, the Time × Groups effect was not statistically significant F (1.26) = 0.005, *p* = 0.94, Eta-squared < 0.001.

**Figure 3:**
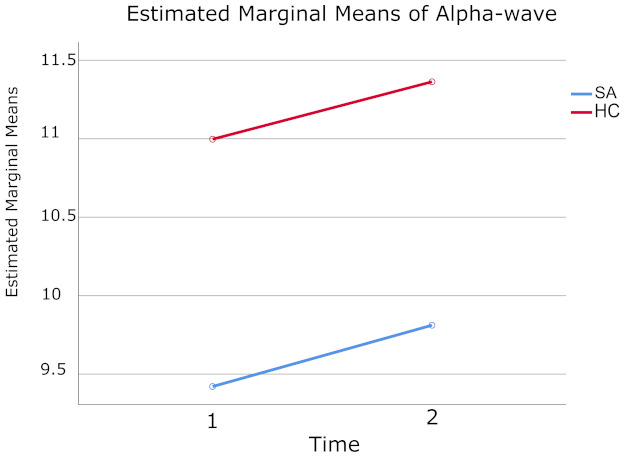
Displayed means for alpha-wave measured at Time 1 (baseline) and Time 2 (after 20 sessions of LTH) for SA (shown in blue color) and HC (shown in red color) groups.

Examination of the cell means indicate that qEEG values of alpha-wave obtained at baseline, and after the LTH program were statistically significant for Time and Groups, but Time × Groups interaction was not statistically significant (see Figure 3). Table 6 contains the descriptive statistics of alpha-waves of SA and HC groups, before and after the complete cycle of LTH.

**Figure 4:**
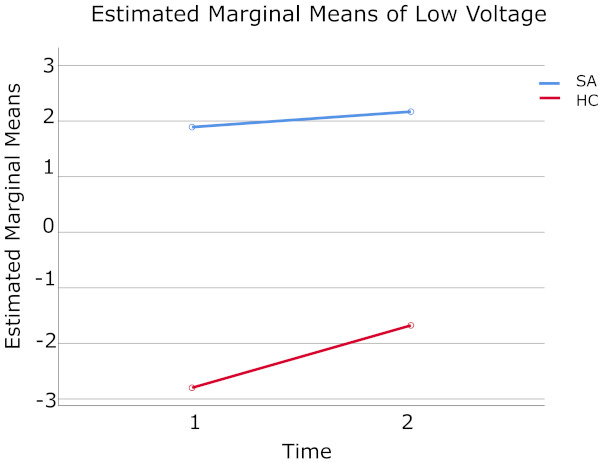
Displayed means for low voltage measured at Time 1 (baseline) and Time 2 (after LTH) for SA (shown in blue color) and HC (shown in red color) groups.

When the values of low voltage were analyzed, the 2 (Time) × 2 (Groups) mixed-model ANOVA reveled that the main effect for Groups was statistically significant F (1.26) = 42.99, *p* < 0.001, Eta-squared = 0.62 (Table 7). Thus, there was a significant overall difference in the value of the low voltage measured for SA (m = 2.03) and HC (m = −2.24) group respectively (Table 8). However, no statistically significant effect for Time was found when SA (m = −0.45) was compared to HC (m = 0.25) group, F (1.26) = 1.54, *p* = 0.23, Eta-squared = 0.06. Nor Time × Groups interaction showed any statistical interest, F (1.26) = 0.56, *p* = 0.46, Eta-squared = 0.02 (see Figure 4).

Examination of the cell means indicate that qEEG values of low voltage obtained at baseline, and after the LTH program were statistically significant only for Groups. Table 9 contains the descriptive statistics of low voltage values before and after the LTH program for both groups (SA and HCs).

**Figure 5:**
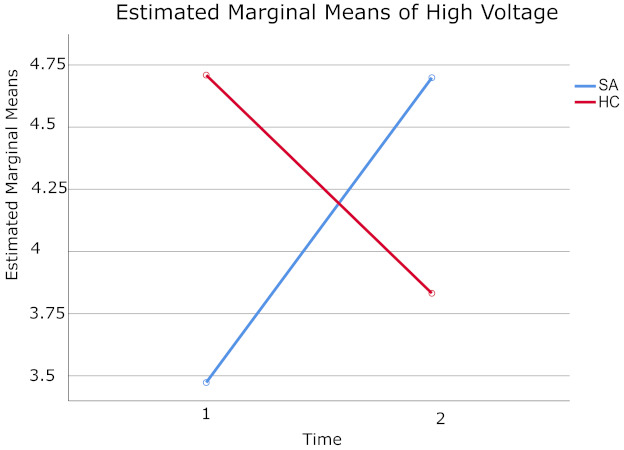
Displayed means for high voltage measured at Time 1 (baseline) and Time 2 (after 20 sessions of LTH) for SA (shown in blue color) and HC (shown in red color) groups.

The 2 (Time) × 2 (Groups) mixed-model ANOVA for high voltage, revealed a significant Time × Groups effect F (1.26) = 8.11, *p* = 0.008, Eta-squared = 0.24 (see Table 10). To analyze its significance, the Wilcoxon test was employed, which revealed a (*p* = 0.04 and 0.01) for SA and HCs respectively. Figure 5 illustrates that while high voltage values for the SA group incremented from Time 1 (m = 3.47) to Time 2 (m = 4.70), they decreased for the HC group (m = 4.71 and 3.83 respectively) (Table 11). While high voltage values measured at baseline (Time 1) were greater for HC group when compared to SA group, the result was inverted when measured after the LTH program (Time 2), where SA high voltage values were higher when compared to HC ones.

However, no statistically significant effect was seen for Groups F (1.26) = 0.17, *p* = 0.68, Eta-squared = 0.007, where the obtained values for SA patients (m = 4.09) were no different form the ones obtained for HC (m = 4.27). Additionally, the effect of Time was not statistically significant either F (1.26) = 0.22, *p*= 0.64, Eta-squared = 0.009. The mean value of high voltage for SA patients (m = 4.09) compared to HCs (m = 4.26) were alike. Examination of the cell means indicate that qEEG values of high voltage obtained at baseline, and after the complete cycle of LTH only presented a statistically significant Time × Groups interaction. Table 12 contains the descriptive statistics of high voltage of SA and HC groups, before and after the LTH program.

A second spectral peak, (theta-wave) was exclusively recorded in SA patients, both, at baseline and after the LTH treatment. The Wilcoxon-test (considering the non-normal distribution of the data analyzed with Shapiro-Wilk test) was used to compare means, with no statistically significant differences between their values (m = 4.62 ± 0.96 and 4.96 ± 1.51 before and after LTH resepctively, where *p* = 0.30).

### 4.3. Spearman Correlation Coefficients

**Figure 6:**
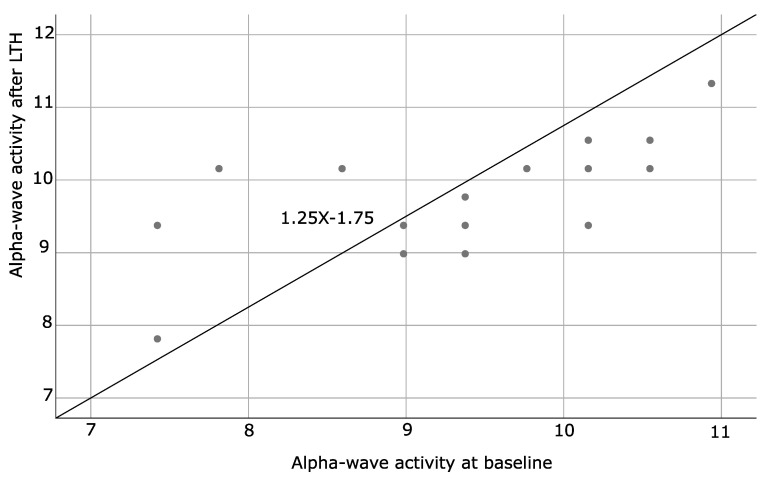
Illustrates the correlation between the alpha-wave activity measured at baseline and after LTH of SA patients.

**Figure 7:**
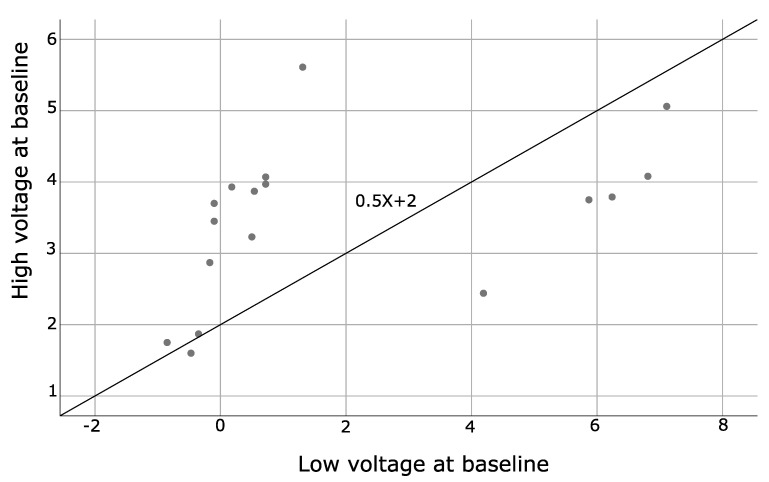
Illustrates the correlation between low and high voltage values measured at baseline of SA patients.

**Figure 8:**
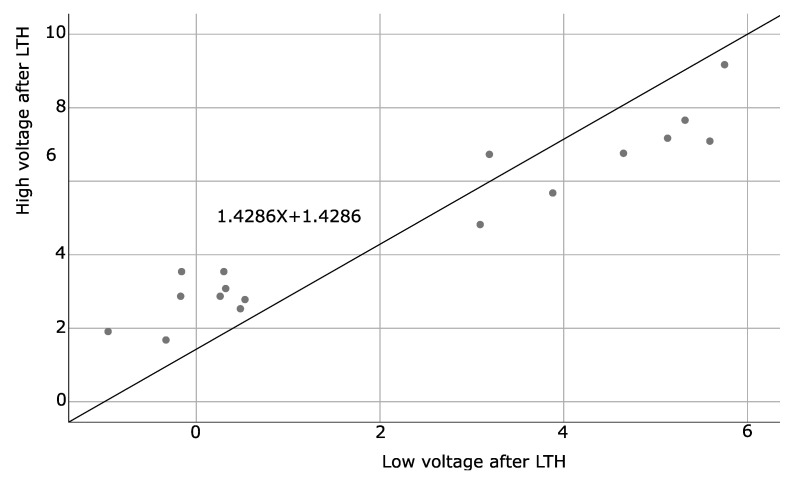
Illustrates the correlation between low and high voltage values measured after LTH of SA patients.

This analysis was used to assess the relationship between two variables based on a monotonic function. SA patients showed positive Spearman correlations for alpha-wave frequency at baseline and after the LTH program (*p* = 0.002), as well as baseline and after LTH low and high voltages (*p* = 0.001 and <0.001 respectively). The results are presented in Table 13 and illustrated by Figure 6, Figure 7 and Figure 8. No statistically significant correlations were found for HCs.

### 4.4. qEEG Differences in the Distribution of Alpha and Theta-Waves across the Brain, at Baseline, and after the Complete Cycle of LTH in SA Patients

When analyzing the baseline qEEG of a healthy patient in the waking-state, alpha waves should be found in the posterior and occipital regions [32]. Table 14 illustrates that only 41.2% of the patients followed this pattern, suggesting an irregular alpha-wave distribution and asymmetric activity pattern, with a predominance in the left hemisphere. By contrast, a better distribution of alpha waves towards the occipital brain regions was seen after the LTH program in 70.6% of the patients (Table 15). A remarkable qEEG finding was the presence of theta waves recorded at baseline and after LTH and its distribution across the brain in SA patients. Theta waves (4–7 Hz) occur primarily during sleep or relaxed wakefulness; their presence in the waking-state is associated with clinical conditions. The distribution of the theta-wave favored the frontal lobe, followed by the occipital and parietal lobes, suggesting that the patients’ conditions may have compromised brain function in the specific cortical areas where theta-wave activity was observed (Table 14 and Table 15). Less theta-wave activity was recorded in the central regions, related to motor areas. After LTH, theta waves were also observed to a limited extent in temporal regions. Nevertheless, its distribution became more homogenous after the LTH treatment, which suggests that enhanced synchronization between hemispheres can be achieved.

### 4.5. qEEG Differences Related to the Brain Coherence at Baseline and after the Complete Cycle of LTH in SA Patients

At baseline, 76.5% of the patients exhibited interhemispheric asynchronicity (absence of brain coherence) (Table 14). By contrast, a better state of interhemispheric synchronicity was found in 76.5% of the patients after LTH (Table 15), indicating the heightened synchronization between the two hemispheres. Hence, light can act as a vector to balance the activity between hemispheres and promote synchronicity across the whole brain. In addition, the state of brain coherence is affected by the wavelength of light transmitted by the filters. After LTH, all patients stimulated with filters transmitting light in the blue spectrum had defined interhemispheric synchronicity of parietal and occipital lobes. Only 62.5% of patients stimulated with filters transmitting light in the red spectrum showed a state of interhemispheric synchronicity after treatment. Even though, no specific brain region could be associated with the asynchronous interhemispheric state of these patients (frontal, temporal, central, parietal, and occipital brain regions were all involved). Nevertheless, the asynchronous state was mostly observed in the left hemisphere. In presence of strabismus, visual and cortical adaptations are not only seen in the amblyopic eye, but in the fellow eye as well. All SA patients were right-handed and 64.7% of them had right eye dominance as well (controlled by the left hemisphere). We contribute the irregular alpha-wave distribution to the hemisphere in charge of the visual processing information. The fellow eye is struggling to compensate for the visual deficiencies presented in the strabismic eye. Therefore, the left hemisphere takes charge of most of the sensorial processing, including eye and hand dominance. Alpha-wave distribution then could be related to the challenges presented to the left hemisphere to maintain the brain functionality at its most. At baseline, relatively increased alpha-wave activity was observed in the left occipital and parietal lobes; increasingly less activity was observed in the right occipital, left central, frontal, and right parietal lobes, respectively. Relatively increased theta-wave activity was observed in the frontal lobe; increasingly less activity was observed in the occipital and right parietal lobes, left parietal lobe, and central regions (associated with sensory and motor functions), respectively. After 20 sessions of LTH, the alpha-wave locates mostly on occipitals, followed by parietals, centrals, and frontals, whereas theta-wave is more present in frontals, followed by occipitals, temporals, parietals, and central regions. It can be concluded that the distribution of the alpha and theta-waves became more homogenous following LTH, indicating the heightened synchronization between the two hemispheres.

### 4.6. qEEG Differences in the Distribution of the Alpha-Wave across the Brain, and the State of Interhemispheric Synchronicity at Baseline, and after the Administration of LTH in HCs

At baseline, alpha waves were mostly found in the occipital lobes, followed by parietals, as expected. Nevertheless, a 27.3% of HCs presented a state of asynchronicity of parieto-occipital lobes at baseline without any clinical manifestation [33]. However, a shift to synchronicity was observed after LTH. (Table 16). To conclude, after LTH, both, the distribution of the alpha-wave and the state of brain coherence, followed normality. The wavelength of light transmitted by the filters could not be associated with changes in the activity of a specific brain region or the state of coherence.

### 4.7. Assessment of the LTH Effect on Clinical Metrics, Such As the Angle of Strabismus, Phoria State, Visual Acuity, Amount of Stereopsis, and Visual Fields

Visual performance was analyzed using parametric and non-parametric tests. The angle of strabismus, visual acuity of SA patients, stereopsis and phoria state were analyzed using the Wilcoxon test, considering the non-normal distribution of the data. Normal data distribution was found for visual fields (for both groups), and visual acuity of HCs. T-paired test was used in this case. The statistical analysis showed that LTH had a great impact on the visual performance of SA patients. It induced enhancements in the visual acuity of both eyes, at far and near distances, increased the amount of stereopsis and 3D perception, decreased the angle of deviation, at both far and near, and enlarged visual fields in response to white, red, green and blue stimulus (see Table 17). LTH destabilized some of the visual abilities of HCs. More specifically, the amount of stereopsis decreased and phoria state was deteriorated, without statistically significant changes in the visual acuity. On the other hand, visual fields become larger in response to all four colored stimuli used. These clinical findings suggest that in patients with strabismus and amblyopia, brain patterns can be actively changed, fostering new visual abilities and improving old ones to secure improved patient outcomes. On the contrary, when no necessary, LTH can act as an aggressor to the visual system when used in healthy population, as seen in HCs.

## 5. Discussion

The present study uses qEEG to obtain and analyze changes in the metrics of brain activity, such as frequencies, voltages, and coherence after a complete cycle (twenty consecutive sessions) of administration of LTH in 17 SA patients, and 11 HCs, aged 8–30 years. The visual performance which included clinical data such as the visual acuity, angle of deviation and phoria state, stereopsis, and dynamic visual fields across stimulation were also analyzed. Being light radiation of between 380–780 nm recommended as a method of retinal stimulation, the visible spectrum of light was chosen for this paper [34]. Two EEG recordings, one at baseline and a second one after the LTH program [17,18], permitted analyzing and comparing data about four parameters associated with the brain activity of the participants. (i) The activity and distribution of the alpha-wave, (ii) the interhemispheric synchronicity representing the state of neural brain coherence, (iii) the presence of a second spectral peak (theta-wave) in SA patients at both recordings, (iv) the anteroposterior gradient indicative of low (anterior brain regions) and high (posterior brain regions) voltages in the brain. qEEG is used as a method of study in the present research, as literature features a very little information about the brain activity of strabismic and amblyopic patients [15] and the influence of LTH on its functionality and the visual system. LTH was administered to the control group to see the effect of the filters used and document it in visually-normal individuals, so that any changes found in the SA group may be considered unique. Moreover, by knowing the mechanism of its function in the control group, simulation could be made for patients with neurodevelopment disorders such as strabismus, amblyopia, or even neurological diseases. The use of filters which selectively transmit short, medium, and long wavelength light has already been useful in traumatic brain injuries and its impact on the alpha-wave activity of healthy population has been recorded using visually evoked potential, being a non-invasive approach. LTH is also a non-invasive treatment, and no side effects have been registered since its introduction in the clinical practice of healthy professionals [17,18].

### 5.1. The Alpha-Wave Activity and Interhemispheric Synchronicity

The alpha-wave activity (8–12 Hz) which is defined by its frequency and spatial topography, has been shown to be reactive to stimuli. The alpha rhythm is the most important component of the EEG signal, and is mostly found in the occipital regions, followed by parietal and posteriors ones. It can reflect a significant thinking information of the human body, representing its cortical functionality [35]. Alpha rhythm is normally present when a subject is mentally inactive, yet alert, with eyes closed and can be easily disrupted by visual attentiveness [33]. Based on the aforementioned, in this research, a particular interest is shown in measuring and monitoring its frequency and symmetry. A 2 (Time) × 2 (Groups) mixed-model ANOVA, revealed that its frequency was higher in HCs when compared to SA patients, at both conditions; (i) baseline, and (ii) after the LTH program. Based on the physiology of the visual system, it could be an indicator of higher levels of visual engagement [36] and enhancement of the integrity and functionality of visual pathways. Additionally, an increase in its frequency after the complete cycle of LTH was found in both groups. Although this incrementation wasn’t statistically significant [37], clinically, EEG oscillations in the alpha band reflect cognitive and memory performance [35,38], and it can derive in an enhanced visual system and its components as well [36]. Several variants of atypical alpha-wave activity, such as frontal, temporal, central, and parietal were observed prior to LS. As different brain areas are associated with specific motor, sensorial or learning and rational cortical activities [39], the spatial distribution of the alpha-wave activity could be related to a detailed cognitive process. After the LTH program, alpha rhythm was prompted towards the occipital and parietals regions, as it is expected in a normal and functional brain. Such a change is associated with a heightened synchronization and communication between hemispheres, as well as a balanced activity of whole brain [32,40]. An interesting fact is that defined interhemispheric synchronicity was seen in all patients stimulated with filters transmitting light in the blue spectrum. On the contrary, an irregular pattern of interhemispheric synchronization and alpha-wave distribution was seen in patients where filters transmitting light in the red spectrum were used. The statistical analysis showed that after LTH, synchronicity was obtained in 62.5% of SA patients stimulated with filters transmitting light in the red spectrum. However, 37.5% of them remained asynchronic. Literature suggests that esotropic patients present deeper and worse sensory-motor visual adaptations than exotropic ones, accompanied by significant changes in the brain network to compensate for their visual deficiencies [5]. Therefore, the reaction to the LTH could be proportional to the amount of the adaptations reached at the visual and cortical level of each patient [25]. The results obtained in the present paper showed that exotropic patients activate interhemispheric synchrony more often than esotropic ones and brain coherence is more probable to be reached in patients with exotropia than patients with esotropia. Therefore, light therapy can be a promising classifier between esotropic and exotropic patients. Research on the monochromatic light of 460 nm, has already shown its impact on alertness, sleep and psychometric measures [41,42], which could explain the positive effect of light in the blue spectrum in the brain activity of SA patients. No significant changes were seen in the distribution of alpha-wave activity and the state of the brain coherence in HCs. These results lead us to the hypothesis that only a dysfunctional visual pathway in need of rehabilitation reacts to a stimulus such as light, in order to overcome its status “quo”. HCs possess already a functional visual system with no clinical manifestation, as well as strong brain network connections. As a consequence, once the light stimulus is off, the brain returns to its previous cortical network organization and functionality. In our previous research, it was demonstrated that a perfect state of synchronization was established in all SA patients when the light stimulus was on, and alpha-wave was distributed to the occipital lobes in all of them, regardless of the wavelength of filters used during the process of stimulation [16]. These results suggest that when the stimulus is on, the brain awakes and becomes active, looking forward to be stimulated. Nonetheless, in this research, is was shown that after the LTH program and once the stimulus is off, such a perfect state of synchronization can not be achieved in all patients, even though important changes and new patterns of organization are recorded in most of them. These results hypothesize that by repeating the LTH program depending on the patients necessity, and introduce new light equipments in the whole treatment program, we could accelerate the process of cortical changes. Undoubtedly, light is a great stimulator which modulates the brain activity; however more research is needed in this field. Beta rhythm appears in the frontal and temporal lobes during excitement, and is the highest brain frequency. In this paper, its frequency and distribution is not considered for the statistical analysis, as not being the purpose of our research [43].

### 5.2. The Anteroposterior Gradient

The anteroposterior gradient defined by high and low voltages captured our interest. The most peculiar finding was the negative value of low voltage measured in HCs, and the positive one seen in SA patients. As expected, the A 2 (Time) × 2 (Groups) mixed-model ANOVA was statistically significant for groups. On the other hand, while high voltage values increased from Time 1 to Time 2 in SA patients, they decreased in HCs, a reason why the A 2 (Time) × 2 (Groups) mixed-model ANOVA revealed an important Time × Group effect; from which it can be suggested that by subjecting HCs to a twenty day program of unnecessary LTH, instability is provoked to the brain network. The brain voltage represents neural activation and is expected to be lower in the anterior regions and higher in posterior ones. Likewise, it must be symmetrical and synchronous across the brain hemispheres. In our research, what should be highlighted, however, is that low voltage took negative values in HCs but positive ones in most of SA patients, both, at baseline and after the complete cycle of administration of LTH. As a consequence, the difference between two measured values (high voltage—low voltage value) was higher in HCs when compared to SA patients. Another scientific data to be distinguished, is the increment of high voltages in SA patients, but its decrease in HCs after the LTH program. In a healthy brain, high voltage is indicative of a greater neural activation and can be an indirect measure of the number of synapses, which in turn defines the neural networks and cortical plasticity [44]. The conclusion drawn then is that in SA patients, LTH promotes a defined anteroposterior voltage gradient, and increases the cortical activity which helps the continuous remodeling of neurosynaptic organization that optimizes the functioning of neural networks. The more signals are sent between neurons, the stronger the connections grow. This phenomenon accounts for why each new experience or event can help the brain to re-wire its physical structure [44]. On the other hand, the decrease in its value in HCs, could indicate that LTH acts as a destabilizing stimulus on the brain activity of a healthy person, as recorded by the qEEG. Based on the aforementioned, it can be suggested that when a specific pattern of light stimulation is offered to a dysfunctional visual pathway, it could trigger new responses in the benefit of brain’s re-wiring process.

### 5.3. The Theta-Wave Activity and Its Distribution

A remarkable scientific finding of the qEEG analysis, is the recording of a second spectral peak (theta-wave, 4–8 Hz), solely in SA patients, at both conditions; (i) baseline, and (ii) after the administration of LTH program, featuring a frontal predominance in most of them. Although there was a non statistically significant increase in its value after the LTH program, clinically it can be translated into a different cognitive and memory performance [35,38]. Normally, theta-waves appears when drowsiness or the central nervous system is in the state of inhibition. Its presence in the waking-state except for indicating a slower neural processing, is also an indirect marker of age [33]. Theta-waves distribution favored the frontal lobe, being its distribution through hemispheres more homogeneous after LTH. Event-related changes indicate that the extent of theta-wave synchronization is positively correlated with the ability to encode new-information [35,38], and oscillations in the alpha and theta band are associated with differences in cognitive and memory performance. It should be highlighted here, the permanence of theta-waves even after the administration of LTH, suggesting that the brain of SA patients maintain the same organization pattern of networks, despite the stimulation provided. Considering that the presence of theta waves in the frontal lobes is generally observed in patients with neurodevelopmental disorders, our findings suggest that strabismus and amblyopia might also be attributable to an aberrant neurodevelopment or dysfunctional cortical maturation, persisting 20 sessions of LTH program. In [16], no theta-wave was recorded when the light stimulus was on, whereas after twenty consecutive session of LTH and once the stimulus is off, theta-wave activity emerges in all patients, with no statistically significant changes in its value. However, enhanced synchronization was confirmed through the qEEG analysis. When it comes to the theta-wave activity, light acts as a powerful brain activator during its use, but in its absence, the same old pattern of organization is recorded. Based on the neurology of the brain activity, theta-wave should normally be absent or very rare during wakefulness, but when present, could indicate a focal, regional, or generalized cortical dysfunction. However, the present study is limited in so far as it did not perform any clinical neuropsychological studies, and potential associations between neurological features and our neuroimaging data could not, therefore, be identified.

### 5.4. Spearman Correlations

Another impressive data of this analysis, were the correlations found between the qEEG metrics, being exclusively to SA patients. A strong positive correlation for the alpha-wave activity before and after the treatment was showed. Low and high voltages were positively related at baseline and after the administration of LTH as well. These results make us hypothesize that the brain organization and activity of patients with strabismus and amblyopia is governed by his own laws. Considering that all patients had primary strabismus, adaptations to their sensorimotor imbalance have already been made to overcome this sensory deficit. We believe that there must be reached such a level of organization to compensate for any kind of visual deficiencies that strabismus may cause [45].

### 5.5. Changes in Visual Metrics

Based on the knowledge that absorption of light by the visual pigments in photoreceptors triggers a cascade of chemical events that increases electrical neural activity, changes in the visual performance of participants were expected to be found after the LTH program. Specifically, significant improvements were seen in all evaluated areas in SA patients, while in HCs, the amount of stereopsis decreased, and the phoria state deteriorated, without affecting visual acuity. Larger dynamic visual fields were measured after light therapy in all participants, being those more evident in SA patients. These results confirm our hypothesis that when an adequate stimulus is provided to a suffering visual system, positive results are obtained, as shown in SA patients. On the contrary, a well organized and functional system can be destabilized when an unnecessary stimulus is given, as seen in HCs. The clinical findings obtained in the present study suggest that when needed, the visual process can be actively changed through LTH, fostering new visual abilities and improving old ones, otherwise, the same stimulation can work as a destabilizer. Considering the presence of neuroplasticity throughout life, with different responses according to age [44], changes can be produced to the visual system as the rest of the sensory and motor modalities. Light therapy should then be implemented as a complementary tool in the treatment of patients with strabismus and amblyopia or those receiving conventional active visual therapy. Furthermore, based on the results obtained during and after a complete cycle of LTH, we strongly believe that more therapies focused on the use of light should be implemented to accelerate the treatment process.

Complementing previous studies on light exposure, our research provides new information about the brain activity and the visual performance of strabismic and amblyopic patients at baseline and after a complete cycle of LTH. This research showed that light is an adequate stimulus to enhance the brain activity and visual abilities of SA patients. The synchronization reached between different brain areas is characteristic to a normative neurophysiological organization and is a target outcome of many therapies related to the child neurodevelopment process [32,46]. Our results might help to inform the future development of clinical treatments and practice. Additionally, considering the multiple projections of the non-visual pathway throughout the brain, the potential of light therapy should be considered in the context of treating SA patients and other neurodevelopmental disorders as well. Finally, this research comes to complement what the above-mentioned studies have shown; that strabismus comes with changes in the whole brain, which are reflected in the brain activity and visual performance; a reason why, LTH should be considered as a potential non-invasive treatment.

## 6. Conclusions

qEEG analysis showed that the brain electrical response and visual performance of patients with SA differs from HCs after the administration of LTH. A higher alpha-wave frequency was recorded for HCs when compared to SA patients, both at baseline and after the LTH program, making the difference statistically significant for Groups (*p* < 0.001) and Time (*p* = 0.04). Negative low voltages were recorded for HCs at both conditions, whereas positive values were recorded for SA patients, making the difference statistically significant for Groups (*p* < 0.001). While high voltage values increased from Time 1 to Time 2 in SA patients, they decreased in HCs, showing a statistically significant difference for Time × Groups (*p* = 0.008). A second spectral peak, theta-wave was exclusively presented in SA patients, both, at baseline and after the LTH treatment, with no statistically significant changes in its frequency from Time 1 to Time 2 (*p* = 0.30). However, its distribution across brain regions became more homogenous, but still maintained a frontal lobe predominance. No theta-wave activity was recorded for HCs. SA patients showed positive Spearman correlations for alpha-wave frequency (*p* = 0.002); baseline and after LTH low and high voltages (*p*= 0.001 and < 0.001 resepctively). No correlations were found for HCs. After the LTH program, the interhemispheric synchronicity incremented from 23.5% to 76.5% and the alpha-wave distribution prompted towards the occipital regions from 41.2% to 70.6% in SA patients. No significant changes were recorded for HCs from Time 1 to Time 2. Significant enhancement in all evaluated visual abilities were recorded in SA patients, where (*p* < 0.001) for visual acuity, stereopsis, esotropia at far, and dynamic visual fields; (*p* = 0.001) for esotropia and exotropia at near, and (*p* = 0.008 and 0.005) for hypertropia at far and near respectively. Deterioration of the phoria state and a decrease in the amount of stereopsis (*p* = 0.001) were found in HCs. No changes were recorded for visual acuity, but larger visual fields were measured after the LTH program (*p* = 0.003). To summarize, LTH produced positive changes in the brain activity and visual performance of SA patients, whereas it deteriorated some of the visual abilities measured in HCs, without affecting the state of brain coherence. The permanent state of theta-waves in SA patients, makes strabismus and amblyopia attributable to an aberrant neurodevelopment process since early ages. LTH then, can only benefit a suffering brain and a visual pathway which needs to be enabled. Its use in healthy population can destabilize the visual system. Considering the importance of the visual system and the large cortical areas involved in processing visual information, LTH gives us the opportunity to modulate the brain activity of SA patients and enhance their visual performance.

## Figures and Tables

**Table 1:** Demographics and clinical measurements at baseline of SA and HC groups.

Parameters	SA Mean ± SD	HCs Mean ± SD	*p*-Value
Male/female	9/8	6/5	-
Age (years)	18.1 ± 10.5	22.3 ± 5.9	0.49
Motor eye dominance	11 R/6 L	9 R/2 L	-
Handedness	17 R	10 R/1 L	-
Angle of esotropia (far/near)	29.0 ± 14.84/27 ± 17.02	-	-
Angle of exotropia (far/near)	12.71 ± 8.30/25.43 ± 12.53	-	-
Angle of hypertropia (far/near)	9.2 ± 3.03/9.2 ± 3.03	-	-
Visual Acuity OD (far/near)	0.32 ± 0.37/0.24 ± 0.36	0.01 ± 0.03/0.03 ± 0.05	0.001/0.004
Visual Acuity OS (far/near)	0.35 ± 0.33/0.2 ± 0.25	0.01 ± 0.03/0.03 ± 0.05	0.001/0.002
Stereopsis	128.8 ± 252.1	25.82 ± 12.81	<0.001

Mann-Whitney test comparing the two groups (*p* < 0.05 represented statistically significant differences). Data shown as mean standard deviation or n. SA, strabismus and amblyopia; HCs, healthy controls; OD, oculus dexter; OI, oculus sinister; R, right; L, left.

**Table 2:** Tests of Between-Subjects Effects for Alpha-Wave.

Source	Type III Sum of Squares	F	Sig.	Partial Eta Square
Intercept	5776.835	4072.123	0.000	0.994
Groups	32.667	23.027	0.000	0.470
Error	36.884			

Transformed Variable: Average.

**Table 3:** Estimates for Groups.

SA and HC Groups	Mean	Std. Error	95% Confidence Interval	
			Lower Bound	Upper BOUND
SA	9.616	0.204	9.196	10.036
HC	11.180	0.254	10.658	11.702

**Table 4:** Tests of Within-Subjects Effects for Alpha-Wave.

	Source	F	Sig.	Partial Eta Squared
Time	Sphericity Assumed	4.542	0.043	0.149
	Greenhouse-Geisser	4.542	0.043	0.149
	Huynh-Feldt	4.542	0.043	0.149
	Lower-bound	4.542	0.043	0.149

**Table 5:** Estimates for time.

Time	Mean	Std. Error	95% Confidence Interval	
			Lower Bound	Upper Bound
1	10.209	0.206	9.786	10.632
2	10.587	0.163	10.253	10.922

1 = alpha-wave measured at baseline, 2 = alpha-wave measured after 20 sessions of LTH.

**Table 6:** Descriptive statics for Alpha-Wave.

	SA and HC Groups	Mean	Std. Deviation	N
Alpha-wave activity at baseline	SA	9.42100	1.103814	17
	HC	10.99718	0.996305	11
	Total	10.04021	1.305439	28
Alpha-wave activity after LTH	SA	9.81153	0.815620	17
	HC	11.36309	0.880818	11
	Total	10.42107	1.130057	28

**Table 7:** Tests of Between-Subjects Effects for Low Voltage.

Source	Type III Sume of Squares	F	Sig.	Partial Eta Square
Intercept	0.574	0.101	0.753	0.004
Groups	243.228	42.986	0.000	0.623
Error	147.116			

Transformed Variable: Average.

**Table 8:** Estimates for Groups.

SA and HC Groups	Mean	Std. Error	95% Confidence Interval	
			Lower Bound	Upper Bound
SA	2.030	0.408	1.191	2.869
HC	−2.237	0.507	−3.280	−1.195

**Table 9:** Descriptive Statistics for Low Voltage.

	SA and HC Groups	Mean	Std. Deviation	N
Low-voltage activity at baseline	SA	1.8912	2.86705	17
	HC	−2.7982	0.66296	11
	Total	0.0489	3.23623	28
Low-voltage activity after LTH	SA	2.1688	2.46504	17
	HC	−1.6764	1.56305	11
	Total	0.6582	2.85709	28

**Table 10:** Tests of Within-Subjects Effects for High Voltage.

Source		F	Sig	Partial Eta Squared
Time x Groups	Sphericity Assumed	8.114	0.008	0.238
	Greenhouse-Geisser	8.114	0.008	0.238
	Huynh-Feldt	8.114	0.008	0.238
	Lower-bound	8.114	0.008	0.238

**Table 11:** SA and HC Groups x Time.

SA and HC Groups	Time	Mean	Std. Error	95% Confidence Interval	
				Lower Bound	Upper Bound
SA	1	3.473	0.224	3.013	3.933
	2	4.699	0.460	3.754	5.643
HC	1	4.709	0.278	4.137	5.281
	2	3.832	0.571	2.658	5.006

**Table 12:** Descriptive Statistics for High voltage.

	SA and HC Groups	Mean	Std. Deviation	N
High voltage activity at baseline	SA	3.4729	1.09514	17
	HC	4.7091	0.54662	11
	Total	3.9586	1.09515	28
High voltage activity after LTH	SA	4.6988	2.33463	17
	HC	3.8318	0.78249	11
	Total	4.3582	1.90857	28

**Table 13:** Spearman correlations of SA group.

			Alpha-Wave Activity after LTH
Spearman’s rho	Alpha-wave activity at baseline	Correlation coefficient	0.692 **
		Sig. (2-tailed)	0.002
			High voltage at baseline
Spearman’s rho	Low voltage at baseline	Correlation coefficient	0.729 **
		Sig. (2-tailed)	0.001
			High voltage after LS
Spearman’s rho	Low voltage after LTH	Correlation coefficient	0.886 **
		Sig. (2-tailed)	<0.001

** Correlation is significant at the 0.01 level (2-tailed); N = 17.

**Table 14:** Distribution of alpha and theta-waves and the state of brain coherence at baseline of SA group.

Patients	Distribution of (α)	Distribution of (θ)	Brain Coherence
001	Occipitals	Frontals	Synchrony
002	Parieto-occipitals	Fronto-centrals, predominating at centrals	Synchrony
003	Occipitals	Frontals and occipitals	Asynchrony of right temporal lobe
004	Frontals and left center-parietal lobe	Left frontal and right parietal lobe	Asynchrony
005	Right fronto-temporal and left parieto-occipital lobe	Frontals	Asynchrony of left temporal lobe
006	Occipitals	Right parieto-occipital lobe	Asynchrony of left temporal lobe
007	Frontals and occipitals	Frontals and centrals	Asynchrony of frontals and parieto-occipitals
008	Center-parietals	Right frontal and right parietal lobe	Synchrony
009	Left parietal lobe	Frontals and occipitals	Asynchrony of left parietal lobe
010	Left center-parietal lobe	Right frontal and right parietal lobe	Asynchrony of left centro-parietal lobe
011	Occipitals	Parieto-occipitals	Asynchrony of fronto-temporal and left parietal lobe
012	Left center-parietal lobe	Left parietal lobe	Asynchrony of left central and parietal lobe
013	Occipitals	Parietals and occipitals	Asynchrony of left frontal lobe
014	Left parieto-occipital lobe	Left parieto-occipital lobe	Asynchrony of left fronto-temporal lobe
015	Occipitals	Occipitals	Asynchrony of fronto-temporals
016	Occipitals	Right frontal lobe	Synchrony of occipitals
017	Parietals	Parietals	Asynchrony of left parietal lobe

Red represents patients who used filters transmitting long-wavelength light (red-orange-yellow) and blue the patients who used filters transmitting low-wavelength light (from bright to dark blue and the combination of grey-blue and violet-blue). Filters transmitting medium-wavelength light (green) are stabilizing filters and were combined either with red or blue filters according to the needs of each patient. EEG recordings were carried-out in the waking-state. (*α*), alpha-wave; (*θ*), theta-wave.

**Table 15:** Distribution of alpha and theta-waves and the state of brain coherence after LTH of SA group.

Patients	Distribution of (α)	Distribution of (θ)	Brain Coherence
001	Occipitals	Frontals	Asynchrony of left temporal lobe
002	Occipitals	Fronto-centrals and centrals	Synchrony
003	Occipitals	Frontals and occipitals	Synchrony of occipitals
004	Occipitals	Fronto-temporals and occipitals	Synchrony of occipitals
005	Occipitals	Frontals	Synchrony of occipitlas
006	Occipitals	Right parieto-occipital lobe	Synchrony of occipitals and parietals
007	Occipitals and parietals	Frontals and centrals	Synchrony of occipitals and parietals
008	Frontals and right centro-parietal lobe	Right frontal and right parietal lobe	Aynchrony of left frontal and centro-parietal lobe
009	Centro-parietals, predominating at centrals	Frontals and occipitals	Synchrony of centro-parietals
010	Occipitals	Right frontal lobe	Asynchrony of left temporal lobe
011	Occipitals and parietals	Fronto-temporals	Synchrony of parietals and occipitals
012	Occipitals	Temporals	Synchrony of occipitals
013	Occipitals	Parietals and occipitals	Asynchrony of left frontal lobe
014	Occipitals	Left frontal, parietal, temporal and occipital lobe	Synchrony of parieto-occipitals
015	Parietals	Frontals	Synchrony of occipitals
016	Occipitals	No theta-wave registered	Synchrony of occipitals
017	Parietals	Parietals	Synchrony parietals

Red represents patients who used filters transmitting long-wavelength light (red-orange-yellow) and blue the patients who used filters transmitting low-wavelength light (from bright to dark blue and the combination of grey-blue and violet-blue). Filters transmitting medium-wavelength light (green) are stabilizing filters and were combined either with red or blue filters according to the needs of each patient. EEG recordings were carried-out in the waking-state. (*α*), alpha-wave; (*θ*), theta-wave.

**Table 16:** Distribution of alpha-wave and the state of brain coherence at baseline and after LTH of HC group.

Patients	Distribution of (α) at Baseline/after LTH	Brain Coherence at Baseline/after LTH
001	Occipitals/Occipitals	Synchrony of occipitals/Synchrony of occipitals
002	Occipitals/Occipitals	Asynchrony of parieto-occipitals/Synchrony of occipitals
003	Occipitals/Occipitals	Synchrony of parieto-occipitals/Synchrony occipitals
004	Parieto-occipitals/Parietals	Asynchrony of parieto-occipitals/Synchrony of occipitals
005	Parieto-occipitals/Occipitals	Asynchrony of parieto-occipitals/Synchrony of occipitals
006	Parietals/Occipitals	Synchrony of parietals/Synchrony of occipitals
007	Parietals/Occipitals	Synchrony of parietals/Synchrony of occipitals
008	Occipitals/Occipitals	Synchrony of parieto-occipitals/Synchrony of occipitals
009	Occipitals/Occipitals	Synchrony of occipitals/Synchrony of occipitals
010	Occipitals/Occipitals	Synchrony of occipitals/Synchrony of occipitlas
011	Occipitals/Occipitals	Synchrony of occipitals/Synchrony of occipitals

Red represents patients who used filters transmitting long-wavelength light (red-orange-yellow) and blue the patients who used filters transmitting low-wavelength light (from bright to dark blue and the combination of grey-blue and violet-blue). Filters transmitting medium-wavelength light (green) are stabilizing filters and were combined either with red or blue filters according to the needs of each patient. EEG recordings were carried out in the waking-state. (*α*), alpha-wave; LTH, light therapy.

**Table 17:** Clinical measurements of SA and HC groups, at baseline and after 20 sessions of LTH.

Parameters	HCs at Baseline	HCs after LTH	*p*-Value	SA at Baseline	SA after LTH	*p*-Value
	Mean ± SD	Mean ± SD		Mean ± SD	Mean ± SD	
VA OD Far	0.01 ± 0.03	0.01 ± 0.03	*p* > 0.05	0.32 ± 0.37	0.16 ± 0.24	*p* < 0.001
VA OD Near	0.03 ± 0.05	0.03 ± 0.05	*p* > 0.05	0.24 ± 0.36	0.12 ± 0.23	*p* < 0.001
VA OI Far	0.01 ± 0.03	0.01 ± 0.03	*p* > 0.05	0.35 ± 0.33	0.2 ± 0.27	*p* < 0.001
VA OI Near	0.03 ± 0.05	0.03 ± 0.05	*p* > 0.05	0.2 ± 0.25	0.14 ± 0.24	*p* < 0.001
Stereopsis	25.82 ± 12.81	25.09 ± 13.99	*p* = 0.001	128.8 ± 252.1	54.2 ± 73.31	*p* < 0.001
ET Far	-	-	-	29.0 ± 14.84	19.13 ± 17.87	*p* < 0.001
ET Near	-	-	-	27.0 ± 17.02	18.13 ± 18.47	*p* = 0.001
XT Far	-	-	-	12.71 ± 8.3	8.14 ± 6.89	*p* = 0.003
XT Near	-	-	-	25.43 ± 12.53	16.57± 10.52	*p* = 0.001
HT Far	-	-	-	9.2 ± 3.03	5.17 ± 3.71	*p* = 0.008
HT Near	-	-	-	9.2 ± 3.03	5.50 ± 3.45	*p* = 0.005
XF Near	12.27 ± 5.69	14.18 ± 6.82	*p* = 0.001	-	-	-
Green OD	15.79 ± 0.6	16.05 ± 0.85	*p* = 0.003	15.38 ± 0.98	17.09 ± 0.39	*p* < 0.001
Green OI	15.94 ± 0.42	16.31 ± 0.46	*p* = 0.003	15.36 ± 0.9	17.25 ± 0.58	*p* < 0.001
Blue OD	24.82 ± 0.81	24.9 ± 1.35	*p* = 0.003	23.87 ± 0.85	25.69 ± 0.65	*p* < 0.001
Blue OI	24.54 ± 0.6	25.14 ± 0.52	*p* = 0.003	23.75 ± 1.2	25.95 ± 0.68	*p* < 0.001
Red OD	23.45 ± 1.31	24.15 ± 1.14	*p* = 0.003	23.02 ± 1.82	25.82 ± 0.67	*p* < 0.001
Red OI	23.71 ± 1.28	24.52 ± 0.65	*p* = 0.003	23.05 ± 1.48	25.98 ± 0.77	*p* < 0.001
White OD	29.55 ± 0.75	29.65 ± 1.39	*p* = 0.003	28.78 ± 1.43	31.04 ± 0.54	*p* < 0.001
White OI	29.39 ± 1.37	29.99 ± 0.97	*p* = 0.003	28.58 ± 1.73	31.09 ± 0.94	*p* < 0.001

T-paired test and Wilcoxon test were used to detect changes between two related samples based on the normality of data distribution. Data shown as mean standard deviation or n. SA, strabismus and amblyopia; HCs, healthy controls; OD, oculus dexter; OI, oculus sinister; ET, esotropia; XT, exotropia; HT, hypertropia.

## Data Availability

The data presented in this study are available on request from the corresponding author. The data are not publicly available due to confidentiality.

## Abbreviations

The following abbreviations are used in this manuscript: LTH, light therapy; SA, strabismus and amblyopia; HCs, healthy controls; qEEG, quantitative electroencephalogram; DBM, digital brain mapping; FFT, fast Fourier transform; CNS, central nervous system; VA, visual acuity; FCFT, functional color field tester; CSO, College of Syntonic Optometry; CI, confidence level; OD, oculus dexter; OI, oculus sinister; R, right; L, left; ET, esotropia; XT, exotropia; HT, hypertropia; (α), alpha-wave; (θ), theta-wave; CONACYT, Consejo Nacional de Ciencia y Teconología.
